# Preparation and Evaluation of Intraperitoneal Long-Acting Oxaliplatin-Loaded Multi-Vesicular Liposomal Depot for Colorectal Cancer Treatment

**DOI:** 10.3390/pharmaceutics12080736

**Published:** 2020-08-05

**Authors:** Sharif Md Abuzar, Eun Jung Park, Yeji Seo, Juseung Lee, Seung Hyuk Baik, Sung-Joo Hwang

**Affiliations:** 1 College of Pharmacy, Yonsei University, 85 Songdogwahak-ro, Yeonsu-gu, Incheon 21983, Korea; sumonzar@gmail.com (S.M.A.); seoyg94@naver.com (Y.S.); ljseung7@gmail.com (J.L.); 2 Yonsei Institute of Pharmaceutical Sciences, Yonsei University, 85 Songdogwahak-ro, Yeonsu-gu, Incheon 21983, Korea; 3 Division of Colon and Rectal Surgery, Department of Surgery, Gangnam Severance Hospital, Yonsei University College of Medicine, Seoul 06273; Korea; camp79@yuhs.ac

**Keywords:** oxaliplatin, multivesicular liposome, depot, sustained release, early postoperative intraperitoneal chemotherapy, colorectal cancer

## Abstract

Colorectal cancer with peritoneal metastasis has a poor prognosis because of inadequate responses to systemic chemotherapy. Cytoreductive surgery followed by intraperitoneal (IP) chemotherapy using oxaliplatin has attracted attention; however, the short half-life of oxaliplatin and its rapid clearance from the peritoneal cavity limit its clinical application. Here, a multivesicular liposomal (MVL) depot of oxaliplatin was prepared for IP administration, with an expected prolonged effect. After optimization, a combination of phospholipids, cholesterol, and triolein was used based on its ability to produce MVL depots of monomodal size distribution (1–20 µm; span 1.99) with high entrapment efficiency (EE) (92.16% ± 2.17%). An initial burst release followed by a long lag phase of drug release was observed for the MVL depots system in vitro. An in vivo pharmacokinetic study mimicking the early postoperative IP chemotherapy regimen in rats showed significantly improved bioavailability, and the mean residence time of oxaliplatin after IP administration revealed that slow and continuous erosion of the MVL particles yielded a sustained drug release. Thus, oxaliplatin-loaded MVL depots presented in this study have potential for use in the treatment of colorectal cancer.

## 1. Introduction

Historically, cancers that spread within the peritoneal cavity were deemed fatal [1,2,3]. Colorectal cancer (CRC), also known as bowel, colon, or rectal cancer, is the development of cancer in the large intestine originating from the colon or rectum. In particular, CRC with peritoneal metastasis shows poor survival and prognosis compared with other metastatic sites [4,5]. Moreover, peritoneal carcinomatosis is believed to be one of the numerous manifestations of CRC identified at the first diagnosis in more than 10% of patients with CRC, resulting in extremely high mortality rates and a median survival of nearly 23.8 months [6]. Systemic chemotherapy has very little effect on improving survival once malignancies have proliferated to the peritoneum; the inadequate blood supply to the peritoneal surface results in low drug stream into tumors [7]. Weissberger (1955) originally introduced the idea of intraperitoneal (IP) chemotherapy for the treatment of peritoneal cancers; later, Dedrick (1978) observed 1–3-mm tissue penetration by several cytotoxic drugs after IP chemotherapy [8]. The underlying principle for the administration of IP chemotherapy is to enable the direct exposure of cytotoxic drugs, along with the tumor cells, without having to rely on the systemic supply to the area.

The two primary approaches for the administration of IP chemotherapy are hyperthermic intraperitoneal chemotherapy (HIPEC) and early postoperative intraperitoneal chemotherapy (EPIC). HIPEC is conducted during surgery for one to two h with an opened or closed abdominal cavity at an optimal temperature of 42–43 °C. As the temperature is increased from 39 °C, synergic heat and drug cytotoxicity begins and tails off at 43 °C [9,10]. The EPIC regimen comprises a postoperative day one administration with continued daily re-administration for five days [11]. During the course, the chemotherapy solution remains in the peritoneal cavity for 23 h and then is drained for one h before re-administration. Although EPIC is one of the promising treatment strategies for patients with CRC and patients with peritoneal carcinomatosis, repeated administration and drain for five days is considered as inconvenient to the patients. Moreover, the chemotherapy solution used in the course contains a high concentration of the drug, which could lead to adverse effects. A decrease in the dose has been suggested, although there are no data to suggest an optimal dose reduction [12]. In this regard, sustained chemotherapy can play a vital role in solving the problems related to re-administration, unwanted toxicity, and dose.

**Figure 1:**
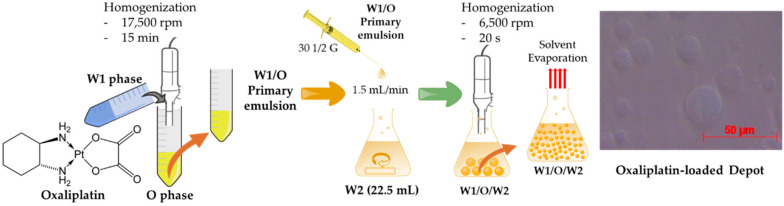
The structure of oxaliplatin, a schematic presentation of the multivesicular liposome (MVL) preparation, and an optical microscopy image of an oxaliplatin-loaded MVL depot.

Platinum-based chemotherapeutic agents are among the most widely used anticancer drugs for the treatment of CRC [13,14]. Despite their success, these drugs have drawbacks, such as poor tumor selectivity and accumulation when administered systemically. Oxaliplatin (Figure 1) is a widely used platinum analog, third-generation alkylating agent for systemic CRC treatment [15,16]. At present, it is a part of both adjuvant and metastatic settings of the standard systemic chemotherapy regimen, FOLFOX (oxaliplatin and 5-fluorouracil with leucovorin) [17]. Oxaliplatin is a cell cycle nonspecific cytotoxic agent that binds to DNA crosslinking (inhibits DNA replication) and is absorbed rapidly after systemic administration [18]. Admitting effectiveness after systemic administration, a recent study on a murine model demonstrated a higher peritoneal tissue concentration of oxaliplatin following IP administration rather than systemic administration. Moreover, an IP administration of oxaliplatin leads to high local concentration, as well as its cytotoxicity improving by hyperthermia [19,20]. With no renal or hepatic toxicity, oxaliplatin is regarded as safe intraperitoneally [18]. While lowering the systemic absorption, the IP administration of oxaliplatin, suggesting a potential decrease in the toxicity (sensory neuropathy), is linked to systemic chemotherapy [21,22]. Therefore, we sought to investigate if the IP administration of oxaliplatin using the EPIC regimen (for sustained chemotherapy) should be researched to determine if a considerable progressive treatment in the disease states of CRC and peritoneal carcinomatosis can be obtained. 

A multivesicular liposome (MVL, or liposomal depot) is a unique system containing particles in internal discontinuous aqueous chambers that is enclosed by a continuous, nonconcentric system of lipid membranes [23,24]. Therefore, MVLs have much larger particle diameters than traditional unilamellar or multilamellar vesicular liposomes [25]. This multivesicular nature also allows a sustained release of the encapsulated drug, and, unlike in unilamellar vesicular liposomes, a single breach in the external membrane of a liposomal depot particle will not result in the complete release of the remaining internal aqueous contents. Previously reported cisplatin-loaded MVL depots provided a sustained drug release suitable for prolonged circulation in a murine carcinoma model [26]. Microparticle and hydrogel-based systems have been used intraperitoneally to control the drug release and prevent the rapid clearance of the drug from the peritoneal cavity [27,28]. We previously reported a higher drug absorption from IP-administered oxaliplatin-loaded poly-(D, L-lactide-co-glycolide) (PLGA) microparticles in crosslinked hydrogels [29]. Early cytoreductive surgery (HIPEC regimen) triggered IP tissue adhesion in more than 50% of cases [30]. IP delivery of these microparticles in the hydrogel effectively reduced the intra-abdominal adhesion while improving the bioavailability and mean residence time. Although the proposed regimen is likely to be an improved therapy for CRC, alternative solutions are required to overcome the problem associated with bulk erosion and the rapid degradation of microspheres and hydrogels, respectively. Several articles reported oxaliplatin-encapsulated liposomes with particle sizes < 200 nm in diameter. These unilamellar and multilamellar liposomes were well-studied for colorectal cancer treatment by systemic administration, and the antitumoral activity was evaluated in vitro and in vivo [31,32,33]. These findings encouraged us to study further for the long-acting IP administration of oxaliplatin using MVL depots.

In this study, oxaliplatin-loaded liposomal depots were prepared by a using the double-emulsion method. To the best of our knowledge, there has been no attempt to prepare oxaliplatin-loaded liposomal depots with sustained release properties to enhance the anticancer efficacy following IP administration. Therefore, the effects of various lipids on the particle size and percentage entrapment efficiency (% EE) were evaluated. In addition, to visualize the inner and outer structure, a confocal microscope was used to assess the morphology of the depots. Furthermore, the depots were evaluated for in vitro and in vivo studies to determine the sustained drug release from the depots and prolonged chemotherapeutic effects after IP MVL depot administrations in rats by using the EPIC regimen.

## 2. Materials and Methods

### 2.1. Materials and Animals

Hydrogenated soybean phosphatidylcholine (HSPC, Lipoid S PC-3; purity 98%) and 1,2-distaeroyl-sn-glycero-3-phosphoehanolamine-conjugated polyethylene glycol-2000 (mPEG-DSPE-2000; purity > 95%) were purchased from Lipoid GmbH (Ludwigshafen, Germany). 1,2-Dierucoyl-sn-glycero-3-phosphocholine (DEPC, Lipoid PC 22:1/22:1; purity > 98%) and 1,2-disteraroyl-sn-glycero-3-phospho-rac-glycerol-sodium salt, (DSPG-Na, Lipoid PG 18:0/18:0; purity > 98%) were a gift from Lipoid GmbH (Ludwigshafen, Germany). β-Cyclodextrin and cholesterol were bought from Sigma Co. Ltd. (St. Louis, MO, USA). Triolein was procured from Kanto Chemical Co. Ltd. (Tokyo, Japan). Sucrose was purchased from OCI Company Ltd. (Seoul, Korea). Dextrose (purity > 98%) was obtained from Samchun Pure Chemical Co. Ltd. (Pyung-tack, Korea). Oxaliplatin was a kind gift from Boryung Pharm (Ansan, Korea). All other chemicals used in this study were reagent grade and utilized without further purification. Milli-Q^®^ water (Millipore 1, Molsheim, France) was used to prepare the formulation and study sample throughout the study.

Male Sprague-Dawley (SD) rats were used in in vivo experiments and were purchased from YoungBio (Seongnam, Republic of Korea). The animals were housed in standard polypropylene cages with stainless-steel lids at 19 °C ± 1 °C and 50% ± 5% relative humidity. The facility was designated as semi-specific pathogen-free with a 12-h light-dark cycle. All experiments were approved by the Institutional Animal Care and Use Committee (IACUC-201910-970-01) at Yonsei University, Seoul, Korea and were performed according to IACUC guidelines.

### 2.2. Preparation of Oxaliplatin-Loaded Liposomal Depot

The oxaliplatin-loaded liposomal depot was prepared in two steps to form a “water-in-oil-in-water” double emulsion. The first step comprised the formation of a “water-in-oil" emulsion. A lipid mixture, containing a combination of phospholipids (HSPC, DEPC, DSPG-NA, and DSPE mPEG-2000); cholesterol; and triolein in various compositions (from Table 1) was dissolved in chloroform. An aqueous solution containing 10 mg of oxaliplatin and 3.5 mM of β-cyclodextrin dissolved in 5% (*w/v*) sucrose (first aqueous solution, W1) was added dropwise to an equal volume (1:1, *v/v*) of lipid solution and homogenized at 17,500 rpm for 15 min to yield a water-in-oil emulsion (primary emulsion). This primary emulsion was emulsified further with 22.5 mL of a 5% dextrose solution (second aqueous solution, W2), resulting in a “water-in-oil-in-water” double emulsion (secondary emulsion). Briefly, a 30-G 1/2 needle was used to reduce the droplet size of the primary emulsion when poured at a flow rate of 1.5 mL/min with continuous stirring at 500 rpm into the second aqueous solution. At this stage, the MVL formed was homogenized immediately at 6000 rpm for 10 s, and 17.5 mL of the second aqueous solution was added further before flushing nitrogen gas over the surface of the emulsion at approximately 35 °C ± 5 °C for 30 min to evaporate the organic solvent. The decreasing turbidity of the MVL suspension indicated a near-complete solvent removal. The resulting MVL suspension was then obtained by centrifugation at 4000 rpm (3220× *g*) for 15 min, washed three times with the 5% dextrose solution, and resuspended again before storage at 4 °C until further evaluation.

### 2.3. Characterization of the Oxaliplatin-Loaded Liposomal Depot

#### 2.3.1. Morphology

The morphology of the MVL depot was evaluated by confocal microscopy (LSM 710, Carl Zeiss, Oberkochen, Germany). Briefly, rhodamine B 1,2-Dihexadecanoyl-sn-Glycero-3-Phosphoethanolamine (Rhodamine-DHPE) and BODIPY^®^ 492/515 were used during MVL preparation to visualize the distribution of the lipid and aqueous phases in the MVL simultaneously through merged images. Rhodamine-DHPE was used as a phospholipid probe with red color, and BODIPY^®^ 492/515 was used as a water-soluble probe with green color. The red and green fluorescent probes were observed at wavelengths of 560–590 nm and 490–530 nm, respectively. Freshly prepared liposomal depots were observed by optical microscopy (Axio scope. A1, Carl Zeiss, Oberkochen, Germany), and microphotographs were captured at 50× magnification.

#### 2.3.2. Particle Size Analysis

The mean particle size and distribution of the MVL depot formulations were analyzed using a Mastersizer 2000 (Malvern Instruments, Malvern, UK) after appropriate dilution with a 5% dextrose solution. The instrument was equipped with red (HeNe gas laser) and blue (LED) light and utilized a laser diffraction technique to determine the particle size range from 0.02–2000 µm. Three repeated studies were carried out for each sample, and the volume mean diameter was plotted. The distribution of the particles was calculated by using Equation (1), and the smaller span values represented a narrow distribution.

(1) $$Span=\frac{D90-D10\;}{D50}\;$$

#### 2.3.3. Drug EE

Drug EE was determined by the centrifugation method. Briefly, 1 mL of the MVL depot suspension was diluted with 10 mL of MeOH and vortex-mixed for 5 min. MVL particles were allowed to dissolve completely, and the resulting solution was filtered using 0.45-µm polytetrafluoroethylene (PTFE) syringe filter followed by appropriate dilution with the mobile phase and analyzed by HPLC. The total content of oxaliplatin (free and entrapped drug) was determined in the depot suspension. A further 1 mL of the depot suspension was used to determine the entrapped oxaliplatin by simply centrifuging at 12,000 rpm for 15 min to separate the liposomal vesicles from the aqueous solution. The upper aqueous phase (supernatant) containing free oxaliplatin was collected, and the concentration was determined by HPLC study. The following Equation (2) was used to calculate the EE:

(2) $$\%\;EE=\frac{Total\;oxaliplatin-Free\;oxaliplatin\;}{Total\;oxaliplatin}\times 100$$

The effects of various lipids and their compositions on %*EE* were also studied.

### 2.4. In Vitro Oxaliplatin Release from the Liposomal Depot

An in vitro drug release study was performed to determine the release profile of an oxaliplatin-loaded MVL depot using 100-kDa dialysis tubes (Float-A-Lyzer^®^ G2, Spectrum Labs, Rancho Dominguez, CA, USA). Briefly, 1 mL of the MVL suspension was transferred into dialysis tubes (previously washed with distilled water, followed by rinsing with a dissolution medium) and then submerged in 20 mL of a dissolution medium (5% dextrose solution) in a capped 50-mL conical tube (SPL Life Science, Gyeonggi-do, Seoul, Korea), which was maintained at 37 °C ± 0.5 °C and stirred at 50 rpm. At predetermined time intervals, 1 mL of the sample was withdrawn from the dissolution medium for determination of the released drug, and the same volume of prewarmed medium was immediately added to the tubes to maintain the sink condition. All experiments were performed in triplicate (*n* = 3), and after appropriate dilution with the mobile phase, collected samples were analyzed by HPLC. The release rate of oxaliplatin from the depot was calculated by the following Equation (3):

(3) $$Cumulative\;oxaliplatin\;release\;\left(\%\right)=\frac{Mt\;}{M0}\times 100\;$$

where *Mt* and *M0* are the measured cumulative oxaliplatin release and initial loaded oxaliplatin, respectively. 

A second approach was also utilized to evaluate the release profile of an oxaliplatin-loaded MVL suspension using rat plasma to mimic the peritoneal environment. Briefly, 20 mL of rat plasma was mixed together with 10 mL of intact MVL suspension. Then, 0.5 mL of the resulting mixture was divided into groups (per time points) and incubated for a certain time under shaking at 100 rpm at 37 °C ± 0.5 °C. At each time point, the incubated samples were placed in polycarbonate centrifuge tubes (Amicon^®^ Ultra-4, Merck Millipore, Gangnam-Gu, Seoul, Korea) to separate the intact MVL in the upper tube through centrifuging (Beckman XL-80 ultracentrifuge, Beckman Coulter, Indianapolis, IN, USA) at 15,000 rpm for 15 min. The released oxaliplatin from the MVL depots was separated from the initial sample and collected in the lower tube. Experiments were performed in triplicate (*n* = 3), and the precipitated clear solution was analyzed by HPLC after appropriate dilution.

### 2.5. In Vivo Oxaliplatin Release in the IP Cavity of SD Rats

In vivo, a pharmacokinetic study was carried out for the prepared MVLs after injection in the IP cavity of SD rats. Oxaliplatin solution was evaluated at the same time for comparison, and rats were treated with a similar regimen to EPIC but with slight modifications. Briefly, 10 male SD rats (4–6 weeks old, 200–210 g) were randomly divided into two groups (*n* = 5 per group) and were anesthetized with isoflurane. In Group 1, an oxaliplatin solution (5 mg/kg) was administered intraperitoneally using a 26-G needle. Similarly, in Group 2, oxaliplatin-loaded MVL (5 mg/kg) was administered intraperitoneally [34,35]. At predetermined time intervals, approximately 0.5 mL of blood was collected from the retroorbital plexus using a heparinized capillary tube and kept in an ice bath. Blood samples were centrifuged at 10,000 rpm (9425× *g*) for 10 min at 4 °C, and the supernatant plasma was obtained and stored at −80 °C until analyzed.

The frozen plasma samples were thawed at 37 °C ± 0.5 °C, and approximately 200 ± 5 mg was weighed and pretreated according to our previous method [29]. Briefly, plasma samples were taken in a lip-type seal vessel (Anton Paar, Graz, Austria), and 6-mL nitric acid was added. Vessels were placed in the microwave machine equipped with a platinum (Pt) sensor (Microwave Reaction System, Multiwave PRO, Anton Paar, Graz, Austria). Samples were heated at 200 °C ± 0.5 °C and 4.0 MPa temperature and pressure, respectively, for 1 h and diluted to 30 mL with deionized water, and the concentrations of ***Pt*** were analyzed using Inductively Coupled Plasma Mass Spectrometry (ICP-MS, NexION 300 D, PerkinElmer, Waltham, MA, USA). Pharmacokinetic parameters—C_max_ (peak concentration), T_max_ (time to peak concentration), AUC_0–120 h_ (area under the curve), AUMC (area under the first moment curve), and MRT (mean residence time)—were calculated according to noncompartmental model analysis.

### 2.6. HPLC Analysis

HPLC analysis was performed using an Agilent 1200 Infinity Series HPLC system (Agilent Technologies, Waldbronn, Germany) to determine the % EE and in vitro oxaliplatin release from MVLs. Briefly, after necessary dilution with the mobile phase, 20 μL of each sample was injected by using an autosampler (Model 1260 ALS, Agilent Technologies, Waldbronn, Germany). The chromatographic analyses were performed through a XTerra™ RPC_18_ column (Waters, Milford, MA, USA; 300 × 3.9 × 5 μm) and detected at 254 nm using an HPLC-UV spectrometer (Agilent 1290 Infinity, Agilent Technologies, Waldbronn, Germany). The mobile phase consisted of a 30:70 (*v/v*) mixture of MeOH and 5-mM sodium-1 heptane sulfonate in water. A flow rate of 1 mL/min of the mobile phase (Model 1260 Quat Pump VL, Agilent Technologies, Waldbronn, Germany) was used. 

### 2.7. Statistical Analysis

Effects of lipid composition on the entrapment efficiency and in vitro and in vivo experimental data were expressed as the mean ± SD of three replicates. Welch’s *t*-test was performed, and a *p*-value of < 0.05 was considered as statistically significant.

### 2.8. Stability

Stability study was performed for the freshly prepared liposomal depots. Samples were incubated at 4 °C and 25 °C in sterile and pyrogen-free tubes (NEST Scientific, Rahway, NJ, USA) covered with aluminum foil for protection from light [36]. Suitable aliquots of the incubated samples were collected at different timepoints (0, 7, 14, and 21 days), and the oxaliplatin concentration was analyzed by HPLC. The percentage of oxaliplatin remaining (% assay) and changes in the % EE were calculated in terms of the initial concentration observed. The samples were studied in triplicate, and the data were expressed as the mean ± SD (*n* = 3). In addition, the degree of aggregation and change in color (if any) were assessed visually before each sampling, and other visible changes were recorded, if any.

## 3. Results

### 3.1. Characterization of the Liposomal Depot

#### 3.1.1. Effect of Lipid Composition 

**Figure 2:**
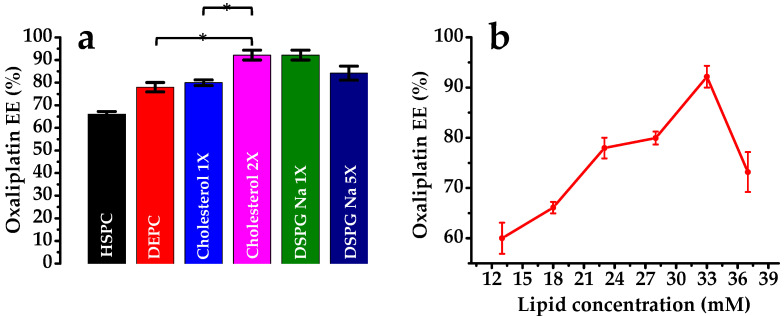
Effects of lipid composition on the entrapment efficiency (% EE) of oxaliplatin on MVLs (**a**) using various lipids and (**b**) using various concentrations (mM) of the total lipids. Data are presented as the mean ± SD (*n* = 3). *Statistically significant. HSPC: hydrogenated soybean phosphatidylcholine, DEPC: 1,2-dierucoyl-sn-glycero-3-phosphocholine, and DSPG Na: 1,2-disteraroyl-sn-glycero-3-phospho-rac-glycerol-sodium salt.

The % EE of oxaliplatin in the liposomal depot prepared using various lipids in variable ratios is shown in Figure 2 and Table 1. The results indicate that, when a combination of lipids is used, entrapment is higher. When HSPC was used, the % EE was only 66.1% ± 1.15%; however, the inclusion of DEPC with HSPC increased the EE to 79.95% ± 1.27%. A higher lipid concentration in the formulation results in increased drug entrapment and/or lower drug leaching [37]. This indicated that the depot formation and stability were dependent on the lipid concentration. DSPE mPEG-2000 was used at a constant mole fraction to maintain the lipid concentration. The % EE was increased up to a lipid concentration of 33 mM; however, it decreased sharply with a further increase in the lipid concentration to 36 mM (Figure 2b). This result was similar to the previous observations of increased EE% with increasing lipid concentrations for small molecules and macromolecules [38]. Moreover, the span value decreased with the increased lipid concentration up to 33 mM, indicating a uniform distribution of the particle size. The ratio of cholesterol to the formulation has a great effect on drug entrapment. When the cholesterol concentration was increased from 5 mM (×) to 10 mM (2×), the drug EE was increased significantly (Figure 2a). The presence of cholesterol between the bilayer of the phospholipids enhanced the rigidity of the nonconcentric network of lipid membranes. Furthermore, a small amount of a triolein is required for the liposome formation; possibly, it becomes a part of the “corners” or “edges” where membranes meet and, thus, stabilizes the membrane boundaries in a manner analogous to planar lipid membranes. When the triolein concentration was increased from 1 mM to 5 mM, the drug entrapment decreased (depots 5 and 6). The reason for this may be that a certain mole fraction of triolein is responsible for the MVL depot formation, and an excess could decrease the drug entrapment and form a triolein-loaded depot [38,39]. Indeed, some of the red vesicular debris in the confocal microphotograph (Section 3.1.3) show agglomerated lipids that probably are collections of triacylglycerols. DSPG-Na is vital for the encapsulation of the aqueous phase. As previously described, the % EE was increased up to a certain concentration of lipids but sharply decreased as the lipid concentration increased further (depots 5 and 7). It is not certain why there is a threshold for the lipid concentration; however, further study on the particle size revealed that an increase in the mole fraction of DSPG-Na also increased the particle size of the MVL depots.

#### 3.1.2. Effect on Particle Size

**Figure 3:**
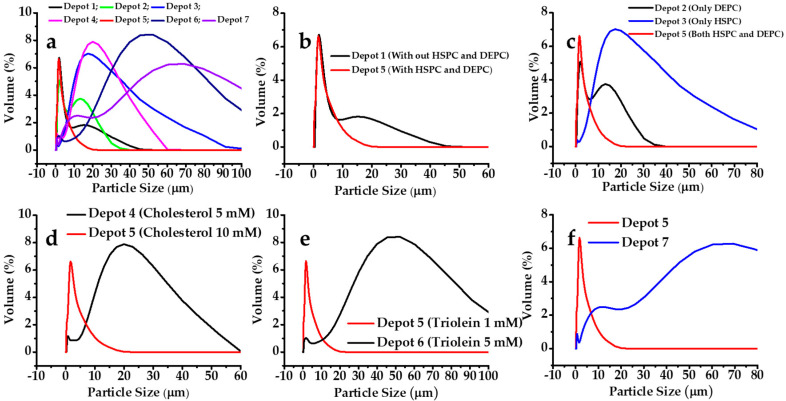
Effects of the lipid composition on the particle size of MVLs: (**a**) depots 1–7; (**b**) depots 1 and 5 (with or without HSPC and DEPC); (**c**) depots 2, 3, and 5 (alone or with a combination of HSPC and DEPC); (**d**) cholesterol 5 and 10 mM; (**e**) triolein 1 and 5 mM; and (**f**) DSPG-Na 1 and 5 mM.

In this study, the volume mean diameter and span value of MVL depots were used to evaluate the particle size and distribution. Among the lipids used in the MVL depot preparations, the combination of HSPC and DEPC showed a lower particle size with uniform distribution. It was observed that an amphipathic lipid with a net neutral charge is required for MVL depot formations. From Figure 3c, it can be seen that the combination of both HSPC and DEPC influenced the particle size. Without an amphipathic lipid, the particle size increased (Figure 3b). Another important factor for MVL depot formations was the presence of a negatively charged lipid. DSPG-Na was used at various ratios. It was observed that only a negatively charge lipid could contribute to depot formation; however, the particle size could be altered. The properties of depots 5 and 7 suggested that an increase in the mole fraction of a negatively charged lipid decreased the drug entrapment, together with an increase in the particle size. The span value was observed as 1.99 and 3.05 for depots 5 and 7, respectively; a lower span value represents a narrow particle size distribution. Therefore, for depot 7, a wide range of particles was observed, with a %EE of 84.19% ± 3.09%. It was explained previously that a small amount of triglyceride (triolein) is vital for depot formation, as it fills up the blank space in corners or edges where membranes meet; however, a larger amount can form triolein-loaded depots. Indeed, when the triolein mole fraction was increased to 5 mM, the particle size was increased. Moreover, a higher cholesterol concentration increased the rigidity of the depot by subsequent incorporation in the lipid bilayer membrane, decreasing the particle size. The findings revealed that depot 5 had monomodal particle size distribution and that 90% of the particles were in the range of 1–20 µm. These results implied that the particles in the oxaliplatin depots had a regular shape and a narrow size distribution.

#### 3.1.3. Morphological Analysis

**Figure 4:**
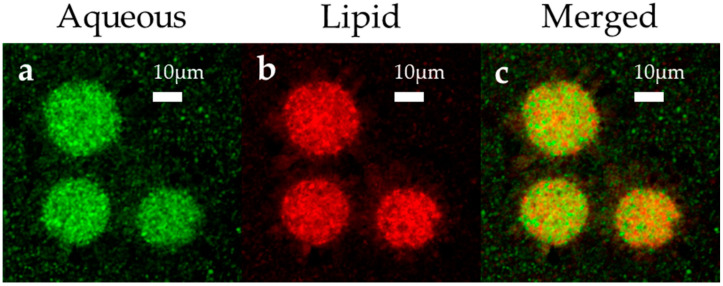
Confocal microscopy images of fluorescent probe-loaded MVL: (**a**) aqueous probe (BODIPY^®^ 492/515), (**b**) lipid probe (rhodamine B 1,2-Dihexadecanoyl-sn-Glycero-3-Phosphoethanolamine, rhodamine-DHPE), and (**c**) merged image (green and red represent the aqueous and lipid phases, respectively). The confocal microphotograph was captured at 60× magnification.

For confocal microscopy, the samples were prepared as for the depot 5 formulation, except that it contained an aqueous probe (BODIPY^®^ 492/515) instead of oxaliplatin and a lipid probe (rhodamine-DHPE) in the lipid solution together in one formulation to visualize and distinguish the distribution of the lipid and the aqueous phases in the depots through merged images. Samples were prepared in the dark, dried overnight on glass slides, and observed at 560–590 nm and 490–530 nm for the red and green probes, respectively. The fluorescent-labeled aqueous phase (Figure 4a; shown in green) was uniformly distributed within the liposomal depots, as expected, as the formation of depots comprises the encapsulation of the aqueous phase within the lipid vesicles formed, and the edge and junctions are filled by the triglycerides. Confocal microscopy allows to observe the aqueous and lipid probe-loaded MLV particles enthusiastically in their native states. Here, the confocal microscopy evaluation expresses the sizes and shapes of the inner and outer vesicles and internal sub-compartments, as previously reported elsewhere [40]. The size distribution of the MVL depots was also visible by confocal fluorescence imaging. As seen in Figure 4a,b, the depots exhibited a general size range of 10–20 μm, and the “honeycomb” structure of the MVL depots was noticeable. The rhodamine-DHPE probes in the lipid layer formed fluorescent fatty acid analogs that effortlessly diffused within the phospholipid layer of the liposomal depots. Moreover, the fluorophore of rhodamine-DHPE remain buried in the hydrophobic internal space of the lipid bilayer membranes and, thus, afforded a detailed outline of the depot’s phospholipid layers. The red fluorescence in Figure 4b shows the lipid layers and is mainly in the outer bilayer of the lipids. These bilayers are responsible for the formation of the vesicles and confer rigidity. The merged image (Figure 4c) reveals the proper distribution of the phases (lipid and aqueous) in these particles, demonstrating an ideal formation of the vesicles. Furthermore, the uniqueness of these liposomal depots is the internal structure, which is clearly illustrated in this figure and is structurally different from that of traditional unilamellar or multilamellar liposomes [41].

### 3.2. In Vitro Oxaliplatin Release

**Figure 5:**
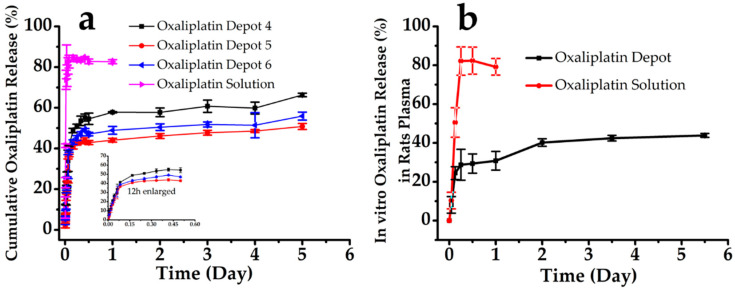
In vitro oxaliplatin release from oxaliplatin-loaded MVL depots and the oxaliplatin solution: (**a**) 5% dextrose solution and (**b**) rats plasma. Study was carried out at 37 ± 0.5 °C, and data are presented as the mean ± SD (*n* = 3).

Oxaliplatin-loaded liposomal depots were evaluated further by an in vitro oxaliplatin release study, and the cumulative drug release (%) versus time (t) plots are shown in Figure 5a. The oxaliplatin solution showed immediate release in a 5% dextrose solution. The three depot formulations (depots 4, 5, and 6) exhibited similar release patterns: an early release phase and a long lag phase, exerting a sustained and more controlled in vitro oxaliplatin release profile [42]. The standard deviations among the triplicate studies of the study groups (*n* = 3) were small, suggesting the outstanding reproducibility of the release performance of the oxaliplatin-loaded depots. However, none of the depots achieved a complete release within the timeframe. For the oxaliplatin solution, a complete release was observed within 6 h; in contrast, oxaliplatin release from the depots was slow. The depot composition influenced the drug release rate. The release rate was slower when the cholesterol content was higher, between 5 mM and 10 mM. Moreover, a higher mole fraction of triolein also reduced the drug release; however, all formulations showed releases over up to five days. 

The oxaliplatin release in rat plasma when incubated at 37 °C is shown in Figure 5b (depot 5). The results were similar to those of the in vitro release study. The oxaliplatin release from the depots in the plasma presents an initial burst release of up to 12 h and then a long lag time, up to two days, followed by a continuous release for at least five days. The findings from the current study were observed to be consistent with earlier reports of in vitro release from depots, where the release of loaded materials was thought to be a sequence of vesicle erosion and diffusion across the vesicle membranes [43,44]. The early burst release within the first 1–6 h was likely to be due to the fact of the freely available drug, either previously dissolved in the bulk medium or rapidly seeped from the depot surface. The lag phase was likely due to the temporary diminution of the superficial drug and, later, the slow diffusion of oxaliplatin across the layer of the lipid barriers. Characteristically, the lag phase showed no substantial drug release following the initial burst release and prior to the secondary release. Thus, the secondary release has been attributed to the ongoing erosion of the MVL by hydrolysis of the lipids [45,46].

Fitting the in vitro oxaliplatin cumulative release percentage (Q) with the Higuchi model equation: Q = kt^1/2^, Weibull equation: LnLn (1/(1 − Q)) = kLn(t − τ)− Lnt_0_, and Hixcon-Crowell equation: (1 − Q)^1/3^ = −kt (Table 2) demonstrated that the depots fitted well with the Hixcon-Crowell and Weibull models.

### 3.3. Pharmacokinetics in Rats

**Figure 6:**
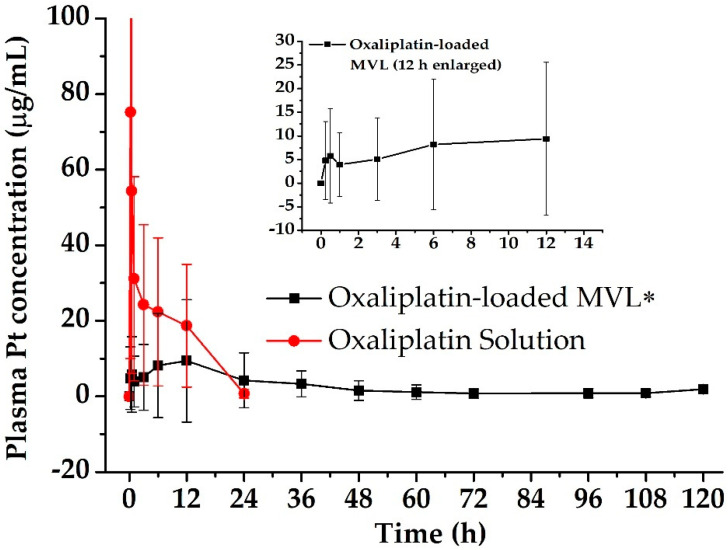
Plasma concentration-time profile of platinum (Pt) in rats after the early postoperative intraperitoneal chemotherapy (EPIC) regimen of the oxaliplatin solution and oxaliplatin-loaded liposomal depots. Data are expressed as the mean ± standard deviation (*n* = 5). * *p* < 0.05.

The EPIC regiment was administered to the rats, and the pharmacokinetic parameters were evaluated for the oxaliplatin-loaded liposomal depots. The mean plasma concentration–time profiles of oxaliplatin after a single dose (5 mg/kg rat) of oxaliplatin solution and oxaliplatin-loaded liposomal depot in rats are shown in Figure 6. The samples were administered by IP injection, with a slight modification to a previous method [29,47]. The IP absorption of the oxaliplatin solution was clearly faster than that of the depot samples (Table 3). For the oxaliplatin solution, the AUC_0–24h_, C_max_, and T_max_ were 412.00 ± 58.54 µg·h/mL, 75.21 ± 8.78 µg/mL, and 0.25 h, respectively. For the oxaliplatin-loaded liposomal depots, the AUC_0–120h_, C_max_, and T_max_ were 316.59 ± 25.29 µg·h/mL, 9.38 ± 16.19 µg/mL, and 12 h, respectively (*p* < 0.05). The C_max_ observed was similar to the earlier reports of hyperthermic IP chemotherapy with 460 mg/m^2^ of a dose to CRC patients [18]; however, MVL depots maintain the concentration for a long duration of time. Moreover, for oxaliplatin-loaded liposomal depots, the AUMC was observed to be three times higher than that for the oxaliplatin solution at 2963.25 ± 315.79 µg·h^2^/mL and 10,690.22 ± 930.67 µg·h^2^/mL, respectively. The MRT of oxaliplatin was increased significantly for the depot formulation owing to the slow release of oxaliplatin from the depots. It was observed that, compared with the oxaliplatin solution, the MRT was four times longer for the oxaliplatin-loaded depots. For the EPIC regimen, the extended residence time of the oxaliplatin is advantageous for the treatment of CRC. MVL depots significantly reduced the C_max_ successively (*p* < 0.05), reducing the IP drug-related toxicity. In addition, a sustained drug release from the depots increased the half-life, resulting in a longer residence of the drug and directly influencing the chemotherapeutic efficacy of the drug.

### 3.4. Stability

**Figure 7:**
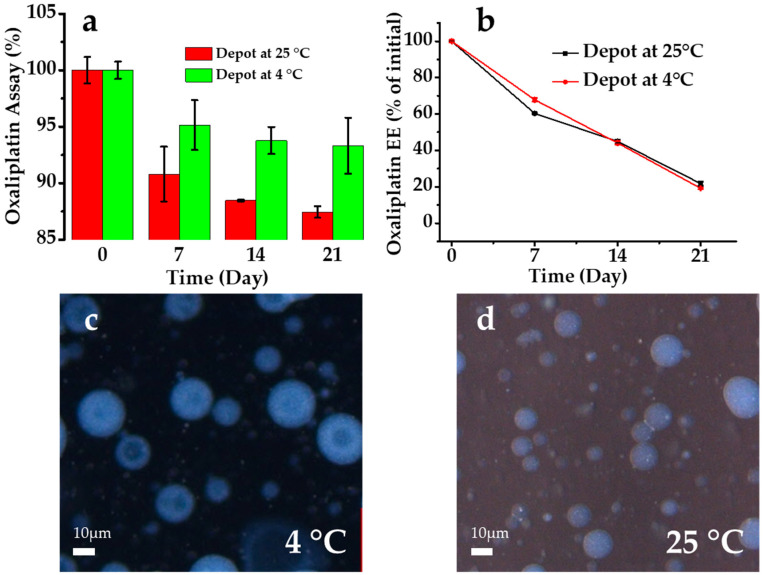
Study of the assay (**a**) and EE (% of initial) (**b**) of oxaliplatin at different time intervals over 3 weeks (stored at 25 °C and 4 °C). Data are expressed as the mean ± standard deviation (*n* = 3). Optical microscopic image presenting the morphology of the MVL depots after 3 weeks of incubation at 4 °C (**c**) and 25 °C (**d**).

A short-term stability study was performed for the oxaliplatin-loaded liposomal depots stored at two different temperature conditions (25 °C and 4 °C). Freshly prepared liposomal depots were evaluated for the percentage of oxaliplatin remaining (% assay) and the % EE after incubation for a predefined time (Figure 7). As drug-leaching and drug degradation were noticeable in the liquid state compared with the solid state or the freeze-dried samples, the experiments were conducted in the liquid state. The data showed that the encapsulation of oxaliplatin in the liposomal depots slightly increased the stability of oxaliplatin. Oxaliplatin is a platinum compound and is unstable in the presence of chloride [48]. Therefore, the laboratory experiments and preparations of the formulations were performed using HPLC-grade water [49]. Temperature plays an important role in oxaliplatin stability. Oxaliplatin degradation was faster for the initial seven days but slowed over time at 4 °C; however, the degradation continued at 25 °C. Moreover, the % EE of the depots was also evaluated after incubation. The high temperature increased the drug release and decreased the % EE; however, a lower storage temperature decreased the drug release. The average drug leakage rate was 50% on day 21 of storage. Similar observations have been reported for several water-soluble drugs with higher drug-leakage rates [40,50]. Liposomal depots are multivesicular liposomes (entrapping hydrophilic drugs in an aqueous phase) containing a large number of aqueous chambers inside, separated by lipid layers. Drugs eventually release from the vesicles by diffusion and the degradation of the lipid layers [51]. There were no significant morphological changes in the depots after storage at 4 °C and 25 °C (Figure 7) and lipid debris was observed in the case of storage at 25 °C after 21 days. This is probably due to the quicker drug leakage from the inner aqueous phase destabilizing the liposomal structure at a higher temperature. Therefore, for a higher stability and longer shelf-life, depots should be freeze-dried and preserved at 4 °C.

## 4. Discussion

In the present study, the MVL depots were prepared, characterized, and evaluated for the sustained IP delivery of oxaliplatin. The lipid vesicular liposomes are classified in two main categories, such as unilamellar (a single bilayer lipid membrane encapsulating an aqueous volume) and multilamellar (numerous concentric membranes, like onions) liposomes. The MVL depot was completely different from these two types and in a class of its own. Depots are multivesicular and connected to each other in a fashion parallel to the blastulae stage of animal embryos. The vesicular wall of the MVL separating an internal aqueous section from another is one single bilayer; therefore, MVL depots have a larger diameter than conventional liposomes [52].

Oxaliplatin is a platinum-based chemotherapeutic agent that has a significant role in the treatment of CRC when given intraperitoneally. Sustained chemotherapy is the key to effective treatment; therefore, oxaliplatin-loaded MVL depots were prepared, characterized, and evaluated for their suitability for IP administration.

Oxaliplatin-loaded MVL particles showed a uniform size distribution in the range of 1–20 µm (span value 1.99), with a high EE% of 92.16% ± 2.17%. As seen in confocal microphotographs, the drug solution-containing aqueous chambers are surrounded by a nonconcentric network of lipid membranes. These nonconcentric lipid membranes maintained a sustained release profile for the entrapped drug. During the storage period, the % EE decreased linearly to allow a controlled release. Surface erosion, the rearrangement of vesicles, and diffusion caused the drug release from the depots [53]. The release study herein was conducted to identify a better approach for the EPIC regimen for postsurgical chemotherapy. Depots that demonstrate a sustained drug release for a prolonged period of time possess the benefits for the EPIC regimen to supply the drug concentration in a less-toxic and less-invasive manner. Moreover, from the postoperative day to consecutive daily therapy for five days includes circulation, drainage, and re-administration of the chemotherapeutics for the EPIC regimen; however, a single administration of sustained oxaliplatin-loaded MVL depots can provide better clinical therapy for the CRC.

An in vivo pharmacokinetic study in rats showed that MVL depots significantly increased the peritoneal absorption of oxaliplatin, with a four-fold higher MRT than that for the solution. This was because of the sustained release of oxaliplatin from the depot; whereas the oxaliplatin solution was absorbed quickly from the peritoneal application site, a shorter MRT was observed for the solution due to rapid clearance. The EPIC regimen contains cytotoxic drugs selected for their cell cycle-specific action that require longer periods of cell contact to achieve cell death [12]. Oxaliplatin is listed as a cell cycle nonspecific cytotoxic agent and, thus, requires appropriate care. High concentrations can be achieved for oxaliplatin locally [35]. Therefore, locally administered oxaliplatin will have similar the synergistic benefits of sustained release, prolonged circulation, and longer contact. Therefore, oxaliplatin-loaded depots will have a lower toxicity and greater chemotherapeutic effect after IP administration. Although oxaliplatin-loaded MVL did not exhibit a degradation of oxaliplatin, as noted in the in vitro release study, a prolonged drug release and site-specific (IP) administration of MVL will definitely provide a good therapeutic effect in patients with CRC. Further studies are required to study the efficacy of oxaliplatin-loaded MVL depots in an in vivo model of CRC.

This study was conducted to formulate, characterize, and evaluate the oxaliplatin-loaded MVL depots; the safety and toxicity of blank MVL depots were not evaluated. The biocompatibility of the MVL depot system needs to be considered. The lipids used in the manufacture of the MVL depots are naturally occurring and are biocompatible. Previous studies have suggested that blank MVL particles do not show any local or systemic toxicity in humans or animals, and there is no “foreign body response” at the injection site after subcutaneous injections [54].

## 5. Conclusions

In this study, we developed liposomal depots containing oxaliplatin by the double-emulsion method. First, various neutral, negative, and amphiphilic lipids were investigated in various ratios; triolein, cholesterol, and a combination of HSPC and DEPC yielded a high % EE and monomodal size distribution for the MVL particles. In addition, the % EE of the liposomal depots was maximized when the lipid concentration was maintained at a particular mole fraction; further increases in the lipid concentration reduced the oxaliplatin entrapment. Confocal microscopy showed the complex inner and outer vesicles of the MVLs in relation to the release behavior of oxaliplatin, which was vital to the insight of the release mechanism of this complex dosage form. The results from our current study deliver excellent knowledge of the formation, arrangement, and release behavior of oxaliplatin-loaded MVLs and their applications in the EPIC regimen. In the future, when the in vivo efficacy (CRC model) is clarified, these MVL depots may be an effective therapeutic option for CRC treatment.

## Figures and Tables

**Table 1:** Compositions of oxaliplatin-loaded multivesicular liposome (MVL) depot formulations. HSPC: hydrogenated soybean phosphatidylcholine, DEPC: 1,2-dierucoyl-sn-glycero-3-phosphocholine, DSPG-Na: 1,2-disteraroyl-sn-glycero-3-phospho-rac-glycerol-sodium salt, DSPE mPEG-2000: 1,2-distaeroyl-sn-glycero-3-phosphoehanolamine-conjugated polyethylene glycol-2000, and EE: entrapment efficiency.

Formulation	Oxaliplatin	β-cyclodextrin	HSPC	DEPC	DSPG-Na	Cholesterol	Triolein	DSPE mPEG-2000	Lipid Conc.	Span	% EE
	mg	mM									
Depot-1	10	3.5	**-**	**-**	1	10	1	1	13	5.69	60.0 ± 3.10
Depot-2			5	-	1	10	1	1	18	4.87	66.1 ± 1.15
Depot-3			-	10	1	10	1	1	23	2.27	77.96 ± 2.05
Depot-4			10	10	1	5	1	1	28	2.08	79.95 ± 1.27
Depot-5			10	10	1	10	1	1	33	1.99	92.16 ± 2.17
Depot-6			10	10	1	10	5	1	37	2.66	73.17 ± 3.97
Depot-7			10	10	5	10	1	1	37	3.05	84.19 ± 3.09

**Table 2:** Fitting equations and correlation coefficients for the in vitro oxaliplatin release in rat plasmas.

	Higuchi	Weibull	Hixcon-Crowell
Oxaliplatin Depot	Q = 16.530t^1/2^ + 12.134 r = 0.876	LnLn(1 /(1 − Q) = 2.065Ln(t) − 7.430 r = 0.928	(1 − Q)^1/3^ = 0.138t + 3.954 r = 0.943
Oxaliplatin Solution	Q = 91.827t^1/2^ + 6.443 r = 0.847	LnLn(1/(1 − Q) = 1.196Ln(t) − 5.175 r = 0.903	(1 − Q)^1/3^ = 2.284t + 2.701 r = 0.897

**Table 3:** In vivo pharmacokinetic parameters after intraperitoneal administration to rats (*n* = 5).

Group	AUC ^1^ (µg·h/mL)	AUMC ^2^ (µg·h^2^/mL)	C_max_^3^ (µg/mL)	T_max_^4^ (h)	MRT ^5^ (h)
Oxaliplatin-Solution	412.00 ± 58.54	2963.25 ± 315.79	75.21 ± 8.78	0.25	7.19
Oxaliplatin-loaded MVL	316.59 ± 25.29	10,690.22 ± 930.67	9.38 ± 16.19	12	29.20

^1^ AUC (area under the curve), ^2^ AUMC (area under the first moment curve), ^3^ C_max_ (peak concentration), ^4^ T_max_ (time to peak concentration), and ^5^ MRT (mean residence time).
